# Caso 4/2020 – Tempo Prolongado (38 Dias) de Extravasamento Pleural Bilateral após Operação Cavopulmonar, Aliviado por Embolização de Vasos Colaterais Sistêmico-Pulmonares, em Cardiopatia Complexa de Criança com 40 Meses

**DOI:** 10.36660/abc.20190488

**Published:** 2020-09-11

**Authors:** Edmar Atik, Raul Arrieta, Fernando Antibas Atik

**Affiliations:** Hospital Sírio Libanês de São Paulo São PauloSP Brasil Hospital Sírio Libanês de São Paulo, São Paulo, SP – Brasil

**Keywords:** Cardiopatias Congênitas/cirurgia, Técnica de Fontan, Dupla Via de Saída, Comunicação Interventricular, Estenose da Valva Pulmonar

## Dados Clínicos

O diagnóstico fetal de anomalia complexa (dupla via de saída das grandes artérias de ventrículo direito, estenose pulmonar acentuada por desvio anterior do septo infundibular, comunicação interventricular trabecular e hipoplasia de ventrículo esquerdo e da valva mitral) foi confirmado logo após o nascimento com hipóxia acentuada, aliviada por prostaglandina E1 e dilatação do canal arterial por *stent* percutâneo. Com o retorno da hipóxia mais acentuada, operação de Glenn bidirecional foi realizada com 9 meses. Boa evolução foi observada até 39 meses, ocasião da operação cavopulmonar total por retorno da hipóxia com saturação de oxigênio de 70%. Usou propranolol e AAS até a última intervenção.

**Exame físico:** bom estado geral, eupneico, cianose acentuada, pulsos normais nos 4 membros. Peso: 16,35 Kg, Alt.: 91 cm, PAMSD: 90 x 60 mmHg, FC: 116 bpm, Sat O_2_: 70%, Hg= 15,5 g, Hct= 55%.

**Precórdio:***ictus cordis* não palpado, sem impulsões sistólicas. Bulhas cardíacas hiperfonéticas, sem sopros. Fígado não palpado. Pulmões limpos.

## Exames Complementares

**Eletrocardiograma:** Ritmo sinusal, com sobrecarga de ventrículo direito.

**Figura 1:**
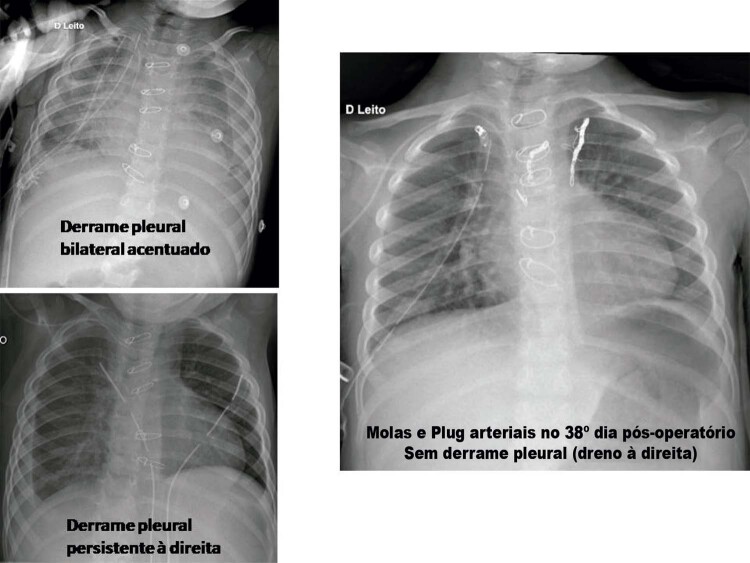
– Radiografias de tórax no pós-operatório da operação cavopulmonar em cardiopatia complexa. As duas da esquerda na expressão dos derrames pleurais e a da direita após a colocação de molas e plug arteriais na demonstração da área cardíaca normal e hipertrófica.

**Radiografia de tórax:** Área cardíaca se mostrava normal com índice cardiotorácico de 0,47. A trama vascular pulmonar era normal ( Figura 1 ).

**Ecocardiograma:***Situs solitus* em levocardia, conexão atrioventricular concordante e conexão ventrículo-arterial do tipo dupla via de saída de ventrículo direito com aorta anterior, comunicação interatrial ampla, comunicação interventricular trabecular não relacionada, com 8 mm de diâmetro e efetivo de 4 mm pela protusão de tecido subvalvar causando fluxo turbulento e com gradiente de pressão interventricular de 22 mmHg, valva tricúspide normal e mitral displásica com folhetos espessados e redundantes. As cordas da valva mitral passam pela CIV em direção à região subvalvar pulmonar. A valva pulmonar é espessa e pequena sem fluxo anterógrado, com atresia funcional. A valva aórtica tem boa abertura, mede 16 mm e a aorta ascendente 17 mm. O ventrículo direito tem 22 mm e hipertrofia da parede de 7 mm. A contratilidade biventricular é normal.

**Cateterismo cardíaco:** Mostrou pressões semelhantes na veia cava superior e na árvore arterial pulmonar, de 10 mmHg. A pressão nos átrios era de 5 mmHg. A angiografia na veia inominada salientou além da boa conexão da veia cava superior na artéria pulmonar direita, árvore pulmonar bem desenvolvida e sem obstruções. No retorno venoso pelas veias pulmonares se notava boa contratilidade ventricular e a anomalia cardíaca bem delineada.

**Diagnóstico clínico:** Dupla via de saída dos grandes vasos de ventrículo direito, estenose pulmonar acentuada por desvio anterior do septo infundibular, comunicação interventricular trabecular e hipoplasia de ventrículo esquerdo e da valva mitral, com Glenn bidirecional e hipóxia acentuada.

**Raciocínio clínico:** Havia elementos clínicos de orientação diagnóstica da malposição arterial pela hiperfonese acentuada das bulhas cardíacas. A estenose pulmonar acentuada era denunciada pela ausência de sopros cardíacos. Não havia indícios clínicos para o diagnóstico da hipoplasia ventricular esquerda, pois a dinâmica funcional se comportava como na dupla via de saída de ventrículo direito com comunicação interventricular e estenose pulmonar. O diagnóstico foi bem estabelecido pela ecocardiografia.

**Diagnóstico diferencial:** Em paciente hipóxico sem sopro expressivo e com bulhas hiperfonéticas, gama imensa de anomalias entram no diagnóstico diferencial. Salientam como principais, a transposição das grandes artérias, ventrículo único direito ou esquerdo e atresia pulmonar com comunicação interventricular. O diagnóstico nessas circunstâncias é sempre estabelecido por imagens ecocardiográficas.

**Figura 2:**
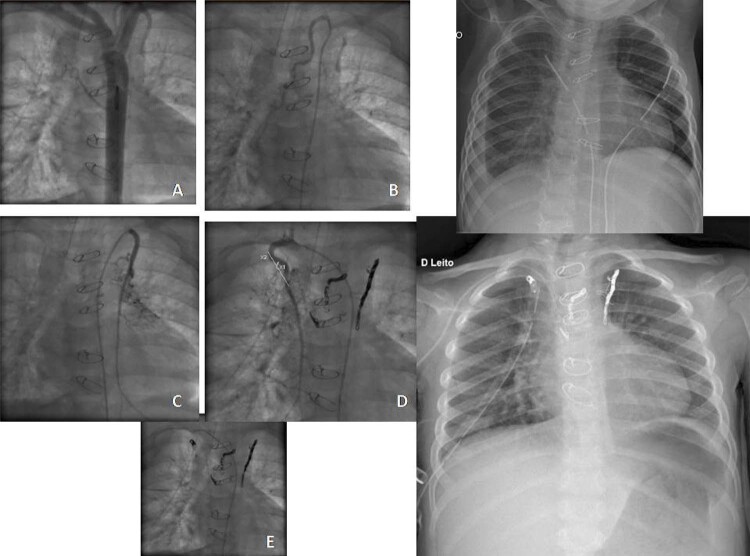
– Colocação de molas e de plug arteriais no fechamento de vasos sistêmico-pulmonares no 38o dia de pós-operatório da operação cavopulmonar. Em A, a fístula a partir da aorta descendente ao pulmão direito, em B e D da artéria torácica interna direita ao pulmão direito, em C da artéria torácica esquerda ao pulmão esquerdo e em E as molas e plug arteriais após todo o procedimento. As radiografias de tórax, prévias (com derrame pleural) e posterior (sem derrame pleural) aos procedimentos.

**Conduta:** Desde o nascimento sabia-se que a conduta mais adequada seria orientada para a operação cavopulmonar total, que se tornou necessária dada a progressão da hipóxia a partir dos três anos de idade. Os dados preliminares antes da cirurgia corretiva funcional pressupunham boa evolução posterior. No entanto, evoluiu com extravasamento exagerado pleural bilateral que perdurou por 38 dias, apesar de tratamento com albumina (6 a 8 g/Kg/dia), furosemida (4 mg/Kg/dia), sildenafila (3 mg/Kg/dia), espironolactona (2 mg/Kg/dia) e restrição hídrica. O derrame pleural bilateral era exagerado e em volume correspondente de 300 a 500 ml por dia e de maneira persistente. Por infecção nos líquidos pleurais, recebeu antibióticos que não solucionaram o problema persistente. No 34^o^ dia pós-operatório, cateterismo cardíaco foi realizado. A pressão média pulmonar era de 16 mmHg. Na angiografia arterial foram detectados 4 pontos discretos de conexão de vasos sistêmico-pulmonares, esparsos pelos 2 pulmões, provenientes das artérias torácicas internas e da aorta descendente. Elas não causavam aumento de saturação nas artérias pulmonares, mas mesmo assim foram fechadas por molas e por *plug* arterial de Amplatzer ( Figura 2 ). Constatou-se, 4 dias após o cateterismo intervencionista, a interrupção das drenagens pleurais e a consequente retirada dos drenos torácicos no 39^o^ dia de pós-operatório. Teve alta hospitalar no 41^o^ dia.

**Comentários:** A evolução pós-operatória da operação cavopulmonar se reveste de muitas surpresas mesmo em pacientes com todos os parâmetros adequados da função ventricular, do tamanho das artérias pulmonares, da pressão e resistência pulmonares, dentre os principais. A formação de fístulas sistêmico-pulmonares parece ocorrer quase de maneira imediata em face da diferença de pressões que se estabelecem entre os sistemas arteriais. Mesmo que não pareçam tão exuberantes, a atuação de embolização das mesmas se faz necessária, principalmente quando o extravasamento pleural se mostra persistente e sem outra causa evidente. No caso presente, acresce-se como outro fator favorável o próprio tempo longo decorrido de pós-operatório que permitiu a esperada acomodação do fluxo pulmonar no contexto da sua árvore arterial e venosa.

Na literatura não encontramos casos com tempo mais prolongado de derrame pleural. Outros procedimentos em casos semelhantes se direcionam à feitura de fenestração, a pleurodese, a ligadura do conduto torácico e o *take-down* do Fontan.^1 , 2^
