# Supplementary material for: Caso 4/2020 – Tempo Prolongado (38 Dias) de Extravasamento Pleural Bilateral após Operação Cavopulmonar, Aliviado por Embolização de Vasos Colaterais Sistêmico-Pulmonares, em Cardiopatia Complexa de Criança com 40 Meses
Source: Arq Bras Cardiol. 2020 Sep 11;115(1 Suppl 1):31–3. [Article in Portuguese] doi: 10.36660/abc.20190488 (PMC8386957; doi:10.36660/abc.20190488)
Supplement: Translation

# Translation: Case 4/2020 – Prolonged Time (38 Days) of Bilateral Pleural Effusion after Cavopulmonary Surgery, Relieved by Embolization of Systemic-Pulmonary Collateral Vessels, in a 40-Month-Old Child with Complex Heart Disease

**Authors:** Edmar Atik, Raul Arrieta, Fernando Antibas Atik

**Affiliations:** Brazil Hospital Sírio Libanês de São Paulo, São Paulo, SP – Brazil

**Keywords:** Heart Defects,Congenital/cirurgia, Fontan Procedure, Double Outlet Right Ventricle, Heart Septal Defects,Ventricular, Pulmonary Valve Stenosis

## Clinical Data

The fetal diagnosis of complex cardiac anomaly (Double-outlet right ventricle, severe pulmonary stenosis due to anterior deviation of the infundibular septum, trabecular interventricular septal defect and hypoplasia of the left ventricle and mitral valve) was confirmed shortly after birth with severe hypoxia, relieved by prostaglandin E1 administration and dilation of the ductus arteriosus by percutaneous stent. With the recurrence of more severe hypoxia, the bidirectional Glenn operation was performed at 9 months of age. Good patient evolution was observed up to 39 months, when total cavopulmonary (Fontan) operation was performed due to the recurrence of hypoxia with 70% oxygen saturation. The patient received propranolol and ASA up to the last intervention.

**Physical examination:** good general status, eupneic, marked cyanosis, normal pulses in the 4 limbs. Weight: 16.35 Kgs, Height: 91 cm, BP: 90 x 60 mm Hg, HR: 116 bpm, O_2_Sat: 70%, Hg = 15.5 g, Hct = 55%.

**Precordium:** nonpalpable ictus cordis, without systolic impulses. Muffled heart sounds, without murmurs. Nonpalpable liver. Clear lungs.

## Complementary Examinations

**Electrocardiogram:** Sinus rhythm, with right ventricular overload.

**Chest x-ray:** Cardiac area was normal with a cardiothoracic index of 0.47. The pulmonary vascular network was normal. ( Figure 1 ).

**Figure 1:** – Chest x-rays in the postoperative period of cavopulmonary surgery in complex heart disease. The two images on the left depict the pleural effusions and the one on the left shows after the placement of arterial coils and plug in the demonstration of the normal and hypertrophic cardiac area.

**Echocardiogram:***situs solitus* in levocardia, concordant atrioventricular connection and Double-outlet right ventricle connected with the anterior aorta, large interatrial septal defect, unrelated trabecular interventricular septal defect, measuring 8 mm in diameter and 4 mm effective area due to subvalvular tissue protrusion causing turbulent flow and with a 22 mmHg interventricular pressure gradient, normal tricuspid valve and dysplastic mitral valve with thickened and redundant leaflets. The mitral valve chordae tendineae passed through the ventricular septal defect (VSD) towards the pulmonary subvalvular region. The pulmonary valve was thick and small, without anterograde flow, with functional atresia. The aortic valve had a good opening and measured 16 mm, while the ascending aorta measured 17 mm. The right ventricle measured 22 mm and the wall hypertrophy was 7 mm. Biventricular contractility was normal.

**Cardiac Catheterization:** It showed similar pressures (10 mm Hg) in the superior vena cava and the pulmonary arterial tree. The pressure in the atria was 5 mmHg. Angiography in the innominate vein highlighted, in addition to the good connection of the superior vena cava in the right pulmonary artery, a well-developed pulmonary tree without obstructions. The venous return through the pulmonary veins showed good ventricular contractility and well-defined cardiac anomaly.

**Clinical Diagnosis:** Double-outlet right ventricle, severe pulmonary stenosis due to anterior deviation of the infundibular septum, trabecular interventricular septal defect and hypoplasia of the left ventricle and mitral valve, with bidirectional Glenn and marked hypoxia.

**Clinical Reasoning:** There were clinical elements leading to an arterial malposition diagnosis due to the markedly muffled heart sounds. Marked pulmonary stenosis was made evident by the absence of heart murmurs. There was no clinical evidence for the diagnosis of left ventricular hypoplasia because the functional dynamics behaved as in the situation of double outlet right ventricle with interventricular septal defect and pulmonary stenosis. The diagnosis was well established by the echocardiography.

**Differential Diagnosis:** In a hypoxic patient without significant murmur and with muffled heart sounds, a wide range of anomalies are included in the differential diagnosis. The main ones are the transposition of the great arteries, a single right or left ventricle and pulmonary atresia with interventricular septal defect. The diagnosis in these circumstances is always established by echocardiographic images.

**Conducts:** It was known from birth that the most adequate approach would be directed at total cavopulmonary surgery, which became necessary given the progression of hypoxia from the age of three. Preliminary data before the functional corrective surgery presumed a good later evolution. However, the patient evolution showed exaggerated bilateral pleural effusion that lasted 38 days, despite treatment with albumin (6 to 8 g / kg / day), furosemide (4 mg / kg / day), sildenafil (3 mg / kg / day) day), spironolactone (2 mg / kg / day) and water restriction. The bilateral pleural effusion was exaggerated and corresponded to a volume of 300 to 500 mL per day, in a persistent manner. Due to infection in pleural fluids, the patient received antibiotics that did not solve the persistent problem. On the 34^th^postoperative day, cardiac catheterization was performed. The mean pulmonary pressure was 16 mmHg. In the arterial angiography, 4 discrete points of systemic-pulmonary vessels’ connection were detected, sparsely distributed in the 2 lungs, coming from the internal thoracic arteries and the descending aorta. They did not cause increased saturation in the pulmonary arteries, but they were still closed by coils and an Amplatzer arterial plug ( Figure 2 ). Four days after the interventionist catheterization, the interruption of pleural drainages was observed, followed by the consequent removal of chest drains on the 39^th^postoperative day. The patient was discharged on the 41^st^postoperative day.

**Figure 2:** – Placement of arterial coils and plug for the closure of systemic-pulmonary vessels on the 38th postoperative day of the cavopulmonary surgery. In A, the fistula from the descending aorta to the right lung, in B and D from the right internal thoracic artery to the right lung, and in C, from the left thoracic artery to the left lung and in E, the arterial coils and plug after the entire procedure. The previous chest x-rays (with pleural effusion) and subsequent one (without pleural effusion), after the procedures.

**Comments:** The postoperative evolution of the cavopulmonary operation has many surprises, even in patients with all the adequate parameters of ventricular function, size of the pulmonary arteries, pulmonary pressure and resistance, among the main ones. The formation of systemic-pulmonary fistulas seems to occur almost immediately due to the difference in pressures that are established between arterial systems. Even if they do not seem so exuberant, their embolization is necessary, especially when the pleural effusion is persistent and there is no other evident cause. In this case, the long post-operative time that allowed for the expected accommodation of the pulmonary flow in the context of its arterial and venous tree counts as another favorable factor. The literature shows no cases with a longer duration of pleural effusion. Other procedures in similar cases include fenestration, pleurodesis, thoracic duct ligation and Fontan takedown^1 , 2^.
